# Hidden in the Spine: Two Cases Illustrating Spondylodiscitis as an Early Red Flag for Mitral Valve Infective Endocarditis

**DOI:** 10.7759/cureus.105490

**Published:** 2026-03-19

**Authors:** Ladislav Kocan, Lucia Mistrikova, Marianna Barbierik Vachalcova, Rudolf Sudzina, Bronislav Vicha, Adrian Kolesar, Stefan Lukacin, Lucia Venglarcikova, Jan Salagovic, Peter Firment, Janka Vašková

**Affiliations:** 1 Department of Anaesthesiology and Intensive Medicine, Faculty of Medicine, Pavol Jozef Šafarik University in Košice, Košice, SVK; 2 Department of Cardiac Surgery, East Slovak Institute for Cardiovascular Diseases, Košice, SVK; 3 1st Department of Cardiology, Faculty of Medicine, Pavol Jozef Šafarik University in Košice, Košice, SVK; 4 Neurology Outpatient Clinic, East Slovak Institute for Cardiovascular Diseases, Košice, SVK; 5 Department of Imaging Techniques, East Slovak Institute for Cardiovascular Diseases, Košice, SVK; 6 Oncology Center, Oncology Clinic, Ziar nad Hronom, SVK; 7 Department of Medical Biology, Faculty of Medicine, Pavol Jozef Šafárik University in Košice, Košice, SVK; 8 Department of Anaesthesiology and Intensive Medicine, J.A. Reiman University Hospital, Presov, SVK; 9 Department of Medical Biology, Faculty of Medicine, Pavol Jozef Šafarik University in Košice, Košice, SVK

**Keywords:** culture-negative endocarditis, infective endocarditis, mitral valve, multidisciplinary management, spinal abscess, spondylodiscitis

## Abstract

Infective endocarditis (IE) remains a severe condition with high morbidity and mortality, often complicated by metastatic infections. Spinal involvement, particularly spondylodiscitis (SD) and epidural or paraspinal abscesses, is increasingly recognized but frequently underdiagnosed. We report two cases of mitral valve IE complicated by SD. A 77-year-old woman with culture-negative IE underwent urgent mitral valve replacement; subsequent magnetic resonance imaging revealed L3-L4 SD and a large right psoas abscess managed conservatively with prolonged antibiotics. A 73-year-old woman presented with cardioembolic stroke and severe mitral regurgitation; spinal MRI showed L4-L5 SD and a long-segment epidural abscess requiring urgent neurosurgical decompression, followed by valve replacement. Both cases highlight diagnostic challenges in culture-negative IE and underscore the need for early multimodality imaging, guideline-directed antimicrobial therapy, and multidisciplinary management. Timely recognition and intervention are essential to prevent irreversible neurological deficits and optimize outcomes.

## Introduction

Infective endocarditis (IE) remains a life-threatening disease with substantial morbidity and mortality despite advances in diagnostics, antimicrobial therapy, and cardiac surgery [1]. Approximately 10-20% of IE cases are culture-negative, most often due to prior antibiotic exposure, which complicates diagnosis and delays appropriate treatment decisions [1]. The most common complications include systemic embolization, acute heart failure, and perivalvular abscess formation. Less frequent but clinically significant are spinal complications, particularly spondylodiscitis (SD) and associated paraspinal or epidural abscesses, which remain challenging to diagnose and manage [2].

SD is an infectious inflammation of the intervertebral disc and adjacent vertebral bodies. Although it accounts for only 3-5% of osteoarticular infections, its prevalence is higher among IE patients, especially those with Staphylococcus aureus or Enterococcus infections [1]. The usual pathophysiological mechanism is hematogenous seeding from valve vegetations. Clinical presentation is often nonspecific - back pain, fever, fatigue - and may mimic degenerative spine disease. Early contrast-enhanced spinal magnetic resonance imaging (MRI) is the diagnostic gold standard, offering high sensitivity and specificity [1,3]. Delayed recognition increases the risk of abscess formation and irreversible neurological damage.

In this study, we present two cases of mitral‑valve IE complicated by SD, each demonstrating a different form of metastatic spinal infection: one with a long‑segment thoracolumbar epidural abscess and the other with a large psoas abscess. Both represented culture‑negative IE, highlighting diagnostic challenges and the importance of timely imaging and guideline‑directed antimicrobial therapy in line with the European Society of Cardiology (ESC) 2023 and Infectious Diseases Society of America (IDSA) 2015 recommendations [4].

Current ESC and IDSA guidelines emphasize early multimodality imaging, multidisciplinary evaluation, and prompt initiation of appropriate therapy, particularly in suspected culture‑negative disease. The 2023 ESC update places greater focus on advanced imaging and a structured team‑based approach, reflecting the increasing complexity of contemporary IE cases.

We hypothesize that early recognition of back pain in IE patients and rapid use of multimodal imaging may lead to earlier detection of metastatic spinal infection and improved outcomes. These cases underscore the need for vigilance regarding spinal involvement and the value of comprehensive, multidisciplinary management in culture‑negative presentations.

## Case presentation

Case 1

**Figure 1:**
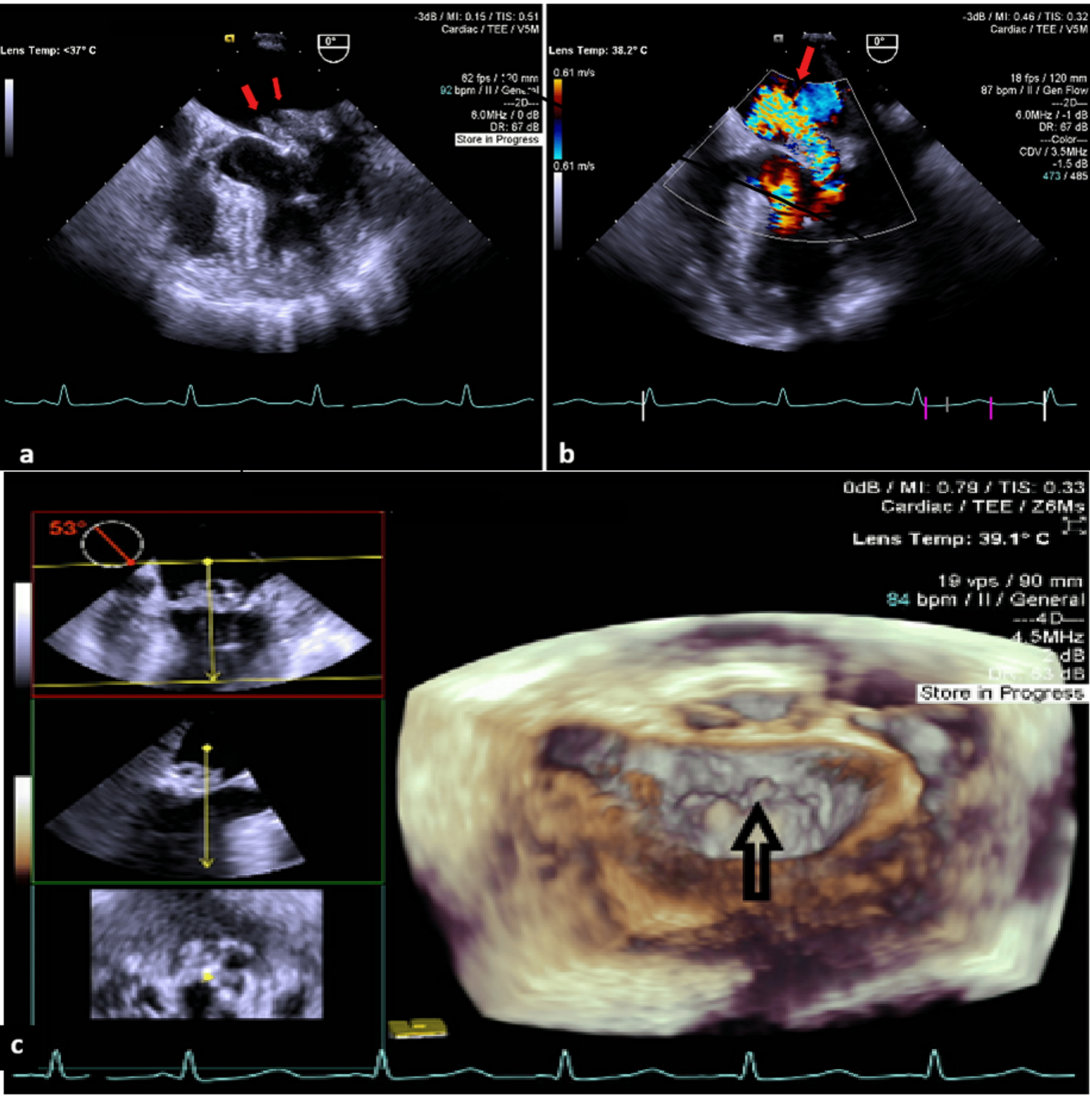
Transesophageal echocardiography views of mitral valve infective endocarditis (A-C). A - Transesophageal echo and visualized multiple vegetations in the area of the posterior leaflet of mitral valve (indicated by an arrow), B - Transesophageal echocardiography and visualized acute severe mitral regurgitation due to infective endocarditis and perforation of posterior leaflet of mitral valve, C - 3D transesophageal echocardiography and visualized vegetations in the area of posterior leaflet of mitral valve (indicated by an arrow)

A 77-year-old woman was transferred from a surgical ward to our cardiothoracic centre with newly diagnosed IE. She had initially been hospitalised for suspected cholangitis and received vancomycin. Transthoracic echocardiography (TTE) demonstrated a large, mobile mitral-valve vegetation with high embolic potential, confirmed by transesophageal echocardiography (TEE) showing multiple vegetations and severe mitral regurgitation (Figure 1).

Ampicillin was administered at 3 g every six hours (total 12 g/day; approximately 170 mg/kg/day), ceftriaxone at 2 g every 12 hours (4 g/day; approximately 57 mg/kg/day), and gentamicin 240 mg once daily (approximately 3.4 mg/kg/day). Prior to transfer, vancomycin had been administered at 15-20 mg/kg per dose with serum trough monitoring while blood cultures and microbiological investigations were pending. On transfer, she was in cardiorespiratory decompensation with generalised oedema (anasarca) and progressive impairment of consciousness. Respiratory failure necessitated escalation from non-invasive ventilation to endotracheal intubation. She was in shock upon admission to our hospital; blood pressure was 85/40 mm Hg with concomitant tachycardia of 113/min. Preoperative coronary angiography revealed no significant obstructive disease. Laboratory tests showed metabolic acidosis, markedly elevated inflammatory markers, moderate anaemia, and moderate thrombocytopenia. Given the life-threatening course, urgent surgery was indicated, and the patient underwent mitral-valve replacement with a bioprosthesis No. 25. The surgical procedure was standard, without complications. The cross-clamp time was 60 minutes, cardiopulmonary bypass time was 88 minutes.

In the early postoperative period she required low-dose vasopressor support; enteral nutrition and early rehabilitation were initiated. Antimicrobial therapy was adjusted according to the results of microbiological testing of the explanted valve. The microbiological examination revealed Klebsiella pneumoniae and Pseudomonas aeruginosa. The chosen effective antibacterial treatment was scheduled for a total duration of six weeks. Intravenous antimicrobial therapy was continued for a total duration of six weeks due to complicated IE with metastatic SD and psoas abscess formation. Perioperative trends in hematologic and inflammatory parameters for both patients are summarized in Table 1.

**Table 1:** Perioperative changes in hematologic and inflammatory parameters from preoperative baseline (PB) to postoperative day (POD) 5 in two patients. PLT – Platelets, WBC – White Blood Cells, CRP – C‑reactive Protein, PCT – Procalcitonin

Patient 1		PB	1POD	2POD	3POD	4POD	5POD
	PLT	139	190	199	270	288	298
	(150-350 x 10^9^/l)						
	WBC	19.9	21.1	10.7	9.4	8.1	9.2
	(4.0-10.0 x 10^9^/l)						
	fibrinogen	4.78	2.8	2.75	1.96	2.1	2.2
	(1.80-3.80 g/l)						
	D-Dimer	1.00	N/A	0.8	N/A	0.5	N/A
	(0.00-0.50 mg/l)						
	PCT	0.10	0.14	0.20	0.12	0.14	0.10
	(0.00-0.10 ug/l)						
	CRP	25.1	16.3	16.1	22.8	10.2	7.5
	(0.5-5.0 mg/l)						
Patient 2		PB	1POD	2POD	3POD	4POD	5POD
	PLT	92	65	150	110	228	215
	(150-350 10^9^/l)						
	WBC	20.1	14.2	7.6	6.1	10.2	12.5
	(4.0-10.0 10^9^/l)						
	fibrinogen	1.4	1.19	3.5	4.2	2.8	.7
	(1.80-3.80 g/l)						
	D-Dimer	1.3		0.7	N/A	1.1	N/A
	(0.00-0.50 mg/l)						
	PCT	1.34	1.30	1.11	0.96	1.30	1.2
	(0.00-0.10 ug/l)						
	CRP	47.7	38.9	31.8	29.9	15.6	10.2
	(0.5-5.0 mg/l)						

**Figure 2:**
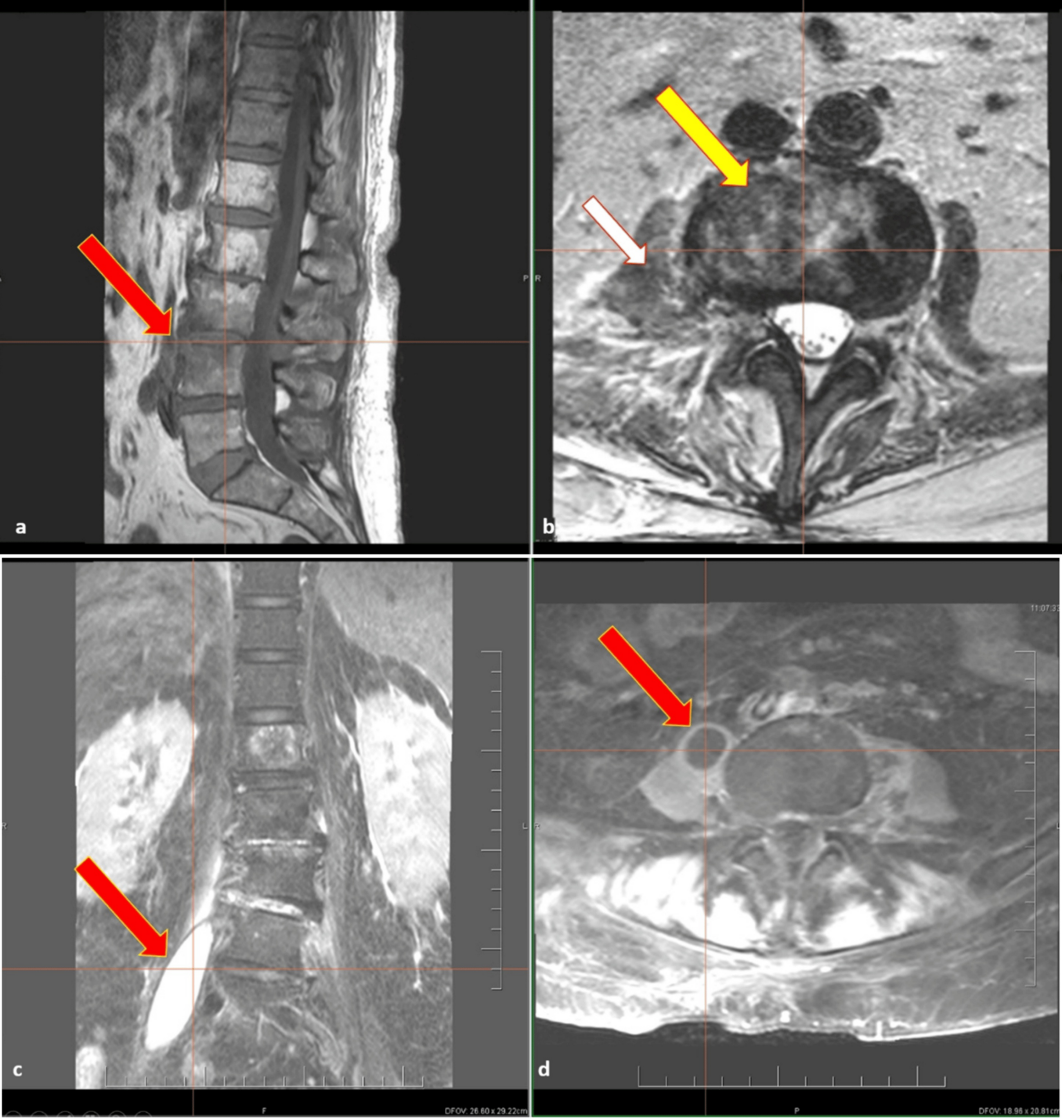
Magnetic resonance imaging of L3–L4 spondylodiscitis with right psoas abscess (A-D). A - Inhomogeneous hypointense areas within the cortical bone and bone marrow of the L3 and L4 vertebral bodies extending to the adjacent endplates, with a hypointense intervertebral disc on sagittal T1-weighted Turbo Spin Echo (TSE) images (red arrow), B - Hyperintense inflammatory changes of the intervertebral disc and adjacent right paravertebral space at the L3/L4 level (yellow arrow), with extension of the inflammatory process into the right psoas muscle on contrast-enhanced axial T1-weighted Spectral Presaturation with Inversion Recovery (SPIR) images (white arrow), C - Hyperintense abscess collection within the right psoas major muscle on T2-weighted Spectral Adiabatic Inversion Recovery (SPAIR) coronal images (red arrow), D - Hyperintense rim of a pyogenic membrane surrounding a hypointense abscess collection in the right psoas muscle on contrast-enhanced axial T1-weighted SPIR images (red arrow). TSE is a fast MRI sequence that provides high‑quality images with shorter scan times compared to conventional spin‑echo techniques. SPIR is a commonly used fat‑suppression method, while SPAIR coronal images employ a selective inversion pulse to suppress fat and enhance the visibility of water‑containing structures such as edema, fluid, or lesions.

Although early mobilisation was attempted, persistent lower-limb motor impairment prompted neurological assessment. Spinal MRI confirmed L3-L4 SD with a large right psoas abscess (Figure 2), findings that guided prolonged antimicrobial therapy and conservative management. Computed tomography (CT; confirmed by MRI) demonstrated SD at L3-L4 with a well-defined hypodense collection in the right psoas muscle measuring approximately 7.5 × 2.5 × 2.0 cm, together with inflammatory changes in the iliacus muscle consistent with an abscessing process. Percutaneous CT-guided drainage and sampling of the psoas collection were attempted but were unsuccessful because of the dense, viscous contents. After multidisciplinary review (cardiac surgery, infectious diseases, neurosurgery, and interventional radiology), a conservative strategy was adopted and surgical evacuation was deferred.

Following infectious disease consultation, antimicrobial therapy was simplified to intravenous linezolid monotherapy 600 mg twice daily for the remaining treatment period. Targeted serology (including Brucella) and an interferon-gamma release assay were negative. Follow-up TEE confirmed a functioning mitral bioprosthesis without residual vegetations; the aortic and tricuspid valves were unchanged and left-ventricular systolic function was preserved. Under conservative management, the patient’s inflammatory markers declined and back pain improved. She remained hospitalised to complete intravenous therapy, monitoring, and rehabilitation, with gradual clinical recovery.

Case 2

**Figure 3:**
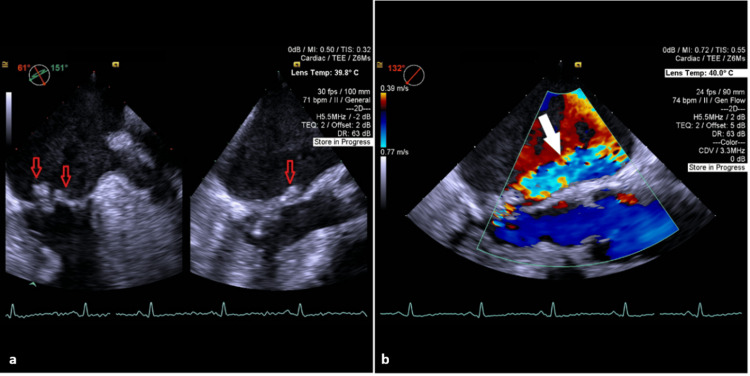
Transesophageal echocardiographic views of mitral valve vegetations and regurgitation (A, B). A - Biplanar projection and visualized vegetations in the area of the posterior leaflet of the mitral valve (indicated by red arrows), B - Transesophageal echo and modified long axis view

A 73-year-old woman with arterial hypertension was urgently transferred to our cardiothoracic centre for definitive management of mitral-valve IE. At the referring hospital she had presented with fever and neurological symptoms; CT brain and CT-angiography (18-19 April 2025) revealed ischaemic lesions in both cerebellar hemispheres and the left pons within the vertebro-basilar territory, suggestive of a cardio-embolic stroke. Initial blood cultures at the referring centre had yielded Staphylococcus lugdunensis, but subsequent blood cultures obtained during admission to our unit were persistently negative, likely influenced by prior antimicrobial therapy. Echocardiography (including transesophageal views) showed extensive destruction of the mitral apparatus with posterior leaflet perforation and vegetations, resulting in severe mitral regurgitation and high embolic risk (Figure 3).

**Figure 4:**
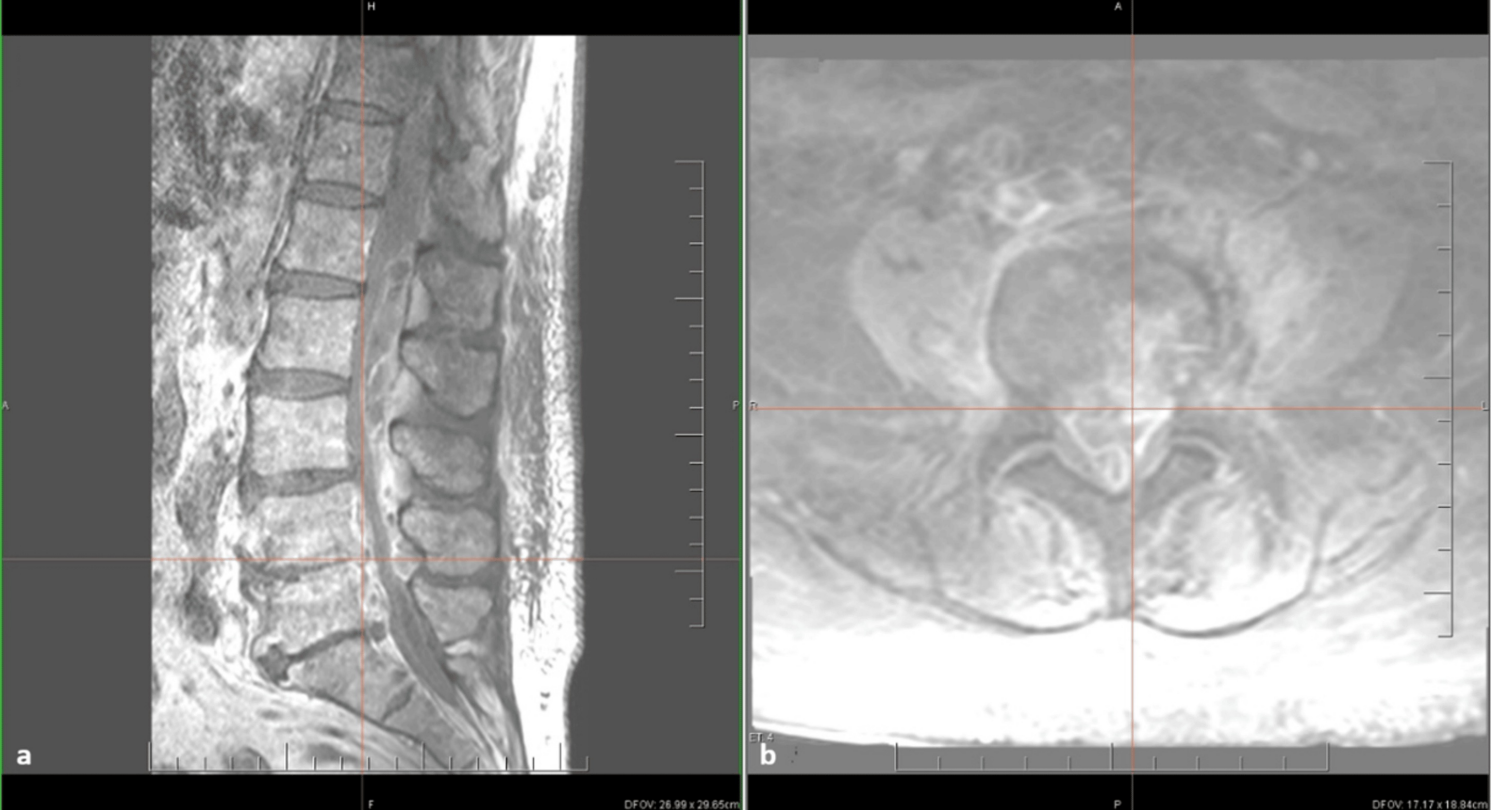
MRI of Epidural Abscess and Spondylodiscitis at L4–L5 (A, B). A - Contrast-enhancing epidural abscess causing compression of the dural sac, with hyperintense signal within the L4/L5 intervertebral disc and adjacent vertebral endplates indicating inflammatory changes (spondylodiscitis), and an epidural fluid collection producing mass effect (sagittal post-contrast T1-weighted Turbo Spin Echo (TSE) images), B - Hyperintense inflammatory changes at the L4/L5 intervertebral disc level (T1-weighted Spectral Presaturation with Inversion Recovery (SPIR)) with visualized mitral regurgitation jet (indicated by a white arrow)

While receiving empirical antibiotics, for a body weight of 58 kg, vancomycin was given at 15-20 mg/kg per dose (approximately 0.9-1.2 g per dose) with serum trough monitoring, she developed severe thoraco-lumbar pain and progressive lower-limb weakness. Contrast-enhanced spinal MRI confirmed advanced L4-L5 SD with a large epidural abscess extending from T11 to L5, causing significant dural sac compression and mass effect (Figure 4). Following multidisciplinary discussion, she underwent urgent neurosurgical decompression with laminectomy and partial evacuation of the epidural collection on 2 May 2025; complete excision of inflammatory tissue was not feasible owing to extensive adherence and multilocularity. Intraoperative abscess cultures were sterile. Because of the severe valvular lesion and ongoing embolic risk, she was prepared for cardiac surgery. She was hemodynamically stable upon admission to our hospital, with a blood pressure of 142/90 mm Hg and heart rate of 80 bpm. Preoperative optimisation included transfusion of blood components, octaplasma and albumin, and correction of severe hypokalaemia. Mitral-valve replacement with a bioprosthesis under cardiopulmonary bypass was performed. The surgical procedure was standard, without complications; however, with longer operating times due to the necessity to perform mitral valve annuloplasty with autologous pericardial patch in order to cover the abscess cavity. The cross-clamp time reached 128 minutes, whereas cardiopulmonary bypass lasted 135 minutes.

The immediate postoperative course was favourable: early extubation, haemodynamic stability without vasopressors, and preserved diuresis. Even though the blood cultures prior to surgery were repeatedly negative, the results of microbiological testing of the explanted valve detected Morganella morganii. Antibiotic treatment was adjusted and continued according to these results. Postoperative transthoracic echocardiography demonstrated preserved left-ventricular systolic function; small bilateral pleural effusions were managed conservatively. Anticoagulation with low-molecular-weight heparin (LMWH) was instituted under anti-Xa monitoring. Neurological recovery was partial, with residual mild paraparesis at transfer.

The patient was stepped down to the cardiac surgery ward in a clinically stable condition to complete guideline-directed antimicrobial therapy and rehabilitation. The identification of Morganella morganii on the explanted valve guided targeted antibiotic adjustment and continuation of therapy for an extended duration of eight weeks of intravenous, followed by two weeks of oral antibiotic therapy, in line with guideline-directed management for complicated IE. Postoperative transthoracic echocardiography demonstrated preserved left-ventricular systolic function; small bilateral pleural effusions were managed conservatively. Perioperative changes in hematologic and inflammatory markers from preoperative baseline to postoperative day 5 for both cases are summarized in Table 1. Postoperatively, anticoagulation with LMWH under anti-Xa monitoring and aggressive rehabilitation were implemented to optimise recovery.

## Discussion

Spinal complications of IE are increasingly recognized as significant contributors to morbidity. Recent series have found vertebral infections (SD) in roughly 8-15% of IE patients [5], particularly in cases caused by Staphylococcus aureus or enterococci. The association of IE with SD is not rare, so clinicians should maintain a high index of suspicion - any IE patient with new back pain warrants prompt evaluation for metastatic spinal infection [5]. Conversely, in patients presenting with spontaneous SD (especially due to S. aureus bacteremia), concurrent endocarditis must be actively ruled out.

Early detection of these linked infections is critical, as delayed diagnosis can lead to paraspinal abscess formation and irreversible neurologic deficits [6]. Guideline-directed diagnostic pathways emphasize rapid, comprehensive evaluation of suspected IE and its complications. The ESC 2023 and IDSA concur that at least three sets of blood cultures should be obtained (from separate venipunctures) before starting antibiotics [4]. Echocardiography is a cornerstone, with TTE followed by TEE in all but the lowest-risk cases, ideally with input from a dedicated Endocarditis Team [5]. In patients with IE who report back or neck pain, guidelines strongly recommend early spinal imaging - preferably MRI with contrast - to detect occult osteomyelitis, discitis, or epidural abscess [4]. MRI is the modality of choice due to its high sensitivity for vertebral and paraspinal infections, while CT can supplement for bony destruction or when MRI is contraindicated [6]. Indeed, ESC guidelines now include spinal lesions among the findings that warrant a multimodality imaging approach in IE [4].

In our cases, the delayed recognition of spinal involvement highlights this point - neurological consultation and spinal MRI were only pursued after persistent back pain and motor deficits were noted, unfortunately after significant abscess formation. Empiric antimicrobial therapy for IE should be initiated promptly after cultures are drawn (unless the patient is hemodynamically unstable, in which case therapy may begin immediately). Broad-spectrum bactericidal coverage is required to address the typical IE pathogens. In acute native-valve endocarditis with a fulminant course, a reasonable empirical regimen is vancomycin plus cefepime, which provides coverage for methicillin-resistant Staphylococcus aureus (MRSA), streptococci, enterococci, and aerobic Gram-negatives [7]. For subacute presentations (indolent course over weeks) without obvious healthcare-associated factors, vancomycin combined with a third-generation cephalosporin (such as ceftriaxone) or an aminopenicillin/beta-lactam inhibitor can be used to cover S. aureus, viridans streptococci, enterococci, HACEK organisms (Haemophilus species, Aggregatibacter species, Cardiobacterium hominis, Eikenella corrodens, and Kingella species), and others. These empiric recommendations align with American Heart Association (AHA)/IDSA guidelines, which suggest vancomycin + cefepime for acute native valve endocarditis (NVE) and vancomycin + ampicillin-sulbactam (or vancomycin + ceftriaxone) for subacute NVE [7].

Once a pathogen is identified, therapy should be narrowed and tailored to susceptibilities. For example, in Enterococcus faecalis endocarditis, a dual beta-lactam regimen of ampicillin plus ceftriaxone for six or more weeks is now preferred over ampicillin-gentamicin, as clinical studies have shown it to be equally effective yet far less nephrotoxic [8]. This approach was applied in our first case: initial broad coverage (ampicillin, gentamicin, and ceftriaxone) was later simplified to targeted therapy [9-11]. In addition to etiological antimicrobial therapy and surgical management, adequate pain control and functional rehabilitation represent essential components of comprehensive care in patients with infective endocarditis complicated by SD. Persistent back pain and neurological impairment may significantly prolong recovery and impair quality of life. Besides standard pharmacological analgesia, adjunctive non-pharmacological modalities may offer additional benefit.

A recent randomized double-blind sham-controlled crossover trial demonstrated significant analgesic efficacy of Rebox electrotherapy in pain management [12]. Although this modality has not been specifically evaluated in infective SD, it may represent a supportive option during prolonged recovery and rehabilitation in selected patients.

## Conclusions

Mitral-valve IE complicated by SD represents a diagnostically challenging and potentially devastating clinical entity. Back pain in patients with IE should never be underestimated and warrants early spinal imaging, preferably contrast-enhanced MRI, to exclude metastatic infection. Culture-negative endocarditis further complicates management and requires a systematic diagnostic approach and guideline-directed empirical antimicrobial therapy.

These cases highlight the importance of early recognition, multimodality imaging, and coordinated multidisciplinary management involving cardiology, cardiac surgery, infectious diseases, neurosurgery, and radiology specialists. Timely surgical intervention combined with appropriate prolonged antimicrobial therapy, typically involving six to eight weeks of intravenous antibiotics followed by an additional two to six months of oral therapy, is crucial to prevent irreversible neurological deficits and improve outcomes.

Comprehensive care should also address functional recovery and adequate pain management, as spinal complications may significantly prolong rehabilitation. Early diagnosis and an integrated therapeutic strategy remain the key determinants of favorable clinical outcomes in this complex patient population.
